# Multiple material need insecurities and severity of psychological distress during the COVID-19 pandemic among women who use drugs

**DOI:** 10.1186/s12889-024-21104-5

**Published:** 2024-12-19

**Authors:** Catherine Tomko, Laura Nicole Sisson, Katherine Haney, Emily Clouse, Natalie Flath, Noya Galai, Katherine C. Smith, Susan G. Sherman

**Affiliations:** 1 Health, Behavior, and Society, Johns Hopkins Bloomberg School of Public Health, 624 N. Broadway St, Baltimore, MD 21205 USA; 2 Mental Health, Johns Hopkins Bloomberg School of Public Health, 624 N. Broadway St, Baltimore, MD 21205 USA; 3 Epidemiology, Johns Hopkins Bloomberg School of Public Health, 615 N. Wolfe St, Baltimore, MD 21205 USA

**Keywords:** COVID, Mental health, Material needs, Women, Substance use, Social determinants of health

## Abstract

**Background:**

The COVID-19 pandemic highlighted the salience of material needs and financial precarity on mental health and distress. Women who use drugs (WWUD) experienced significant mental distress and multiple material need insecurities before the pandemic. However, research is limited on the nature of these insecurities during the pandemic despite both material scarcity and mental distress placing WWUD at greater risk of drug-related harms such as overdose. We aim to characterize material need insecurities and their associations with level of mental distress among a sample of WWUD in the United States during the COVID pandemic.

**Methods:**

*N* = 227 WWUD (i.e., non-medical use of opioids, crack or powdered cocaine at least three times in the past three months) were recruited in Baltimore, Maryland between August 2021-December 2022. We assessed participants’ challenges (i.e., none, minor, major challenge) in accessing five critical material needs during the pandemic: housing; food security; clean, potable water; transportation; and bathroom facilities. The number of major challenges was summed and dichotomized as minimal (0–1) challenges vs. multiple (2+) challenges. The outcome was severity of mental distress, measured by the Kessler-6 and categorized into no/mild, moderate, or severe distress based on validated cut-points. Multinomial logistic regression models were used to explore differences between mental distress severity groups.

**Results:**

36% of the sample reported symptoms of moderate psychological distress and 39% severe distress. Nearly half (43%) reported multiple material need insecurities during COVID. Compared to those who reported minimal material need insecurities, participants with multiple insecurities were 3.25 (95% CI = 1.42–7.45) and 1.96 (95% CI = 0.97–3.95) times more likely to report severe psychological distress compared to no/mild distress or moderate distress, respectively. Unmet mental health needs increased risk of severe distress compared to no/mild 3.44 (95% CI = 1.48–7.97) or moderate 3.62 (95% CI = 1.75–7.49) distress.

**Conclusions:**

WWUD experienced a substantial burden of multiple material needs during the pandemic which were associated with elevated levels of mental distress. Results speak to the need to advance mental health equity by addressing access to material needs and mental healthcare for marginalized populations, particularly during health emergencies that threaten already-precarious social safety nets and healthcare infrastructure.

## Background

The public health emergency of the COVID-19 pandemic disrupted medical, economic, social, and other sectors of life and negatively affected mental health across the globe. In the United States (U.S.), fear of infection, uncertainty about the pandemic’s trajectory, and isolating COVID mitigation policies like stay-at-home orders andquarantines were significant stressors [1]. A recent meta-analysis of mental disorders experienced during the pandemic estimated point prevalence of anxiety, depression, and general psychological distress at 21%, 18%, and 13%, respectively, markedly higher than pre-pandemic rates of mental disorders [2].

COVID placed into stark relief the importance of material needs and financial precarity on mental health sequelae [3, 4]. A recent theoretical framework by Whittle & colleagues explicates the potential pathways through which public policy shapes individual-level material needs and subsequent health outcomes including mental health [5]. The Precarity in Health framework suggests that a rise in insecure labor markets and shrinking social welfare investment in Western European and North American countries can lead to precarity in health for vulnerable populations by facilitating multiple material need insecurities [5, 6]. Specifically, the framework considers insecurities in income, housing, food, and healthcare beyond extreme deprivation including sufficiency, quality, uncertainty, social acceptability, to show that extreme deprivation is only one facet of material need. Multiple, intersecting material needs are theorized to contribute to internalization, self-blame, and shame; anxiety may arise from the uncertainty of public institutions offering robust support in times of economic crisis [5]. Ultimately, public policy and individual-level needs work in tandem to produce psychosocial harms including stress, anxiety, and depressive symptoms. These factors all compounded in the U.S. during the COVID-19 Federal Emergency shutdown.

In research conducted prior to the pandemic, material needs and other insecurities in the social determinants of health (SDoH) have been linked to poorer mental health outcomes and onset of mental illness often due to increases in anxiety and stress load [7, 8]. COVID-19 excerbated the impacts of financial precarity and mental health distress. An estimated 40% of U.S. adults reported a financial stressor related to the pandemic [9]. Unemployment, wage decreases, and unsafe working conditions for essential workers, combined with global macroeconomic downturns, created or exacerbated anxiety and depression in the general population, with the greatest impact among low-income populations [10, 11]. Material need insecurities occurring during the pandemic showed similar associations with deleterious mental health outcomes as pre-pandemic, though the scope and severity of mental distress or illness often increased [2]. Experiencing multiple COVID-related material need stressors (e.g., housing insecurity, diminished wages) was associated with increased odds of probable depression, generalized anxiety disorder, and more severe post-traumatic stress symptoms in a nationally-representative sample of US adults [9, 12].

The pandemic highlighted systemic inequities that inherently affect communities already margalized within the social, economic, and health ecosystem. Research has shown that women were one of the populations for whom mental health was most affected during the COVID era [2, 13]. Women who use drugs (WWUD) are one marginalized population that experienced significant mental distress and multiple material need insecurities before the pandemic began [14, 15], though there is limited research about how these challenges manifested during the pandemic. The relationship between substance use and poor mental health is bidirectional, with individuals self-medicating with drugs and alcohol to cope with their mental health and substance use (especially withdrawal) intensifying feelings of depression or anxiety, for example [16]. Nevertheless, WWUD face numerous threats to their mental health and wellbeing stemming from economic and social disenfranchisement [17]. For example, accessing employment outside of the street economy can be difficult while actively using drugs (due to occupational drug testing) or due to prior criminal legal involvement. Women and people of color (especially Black individuals in the U.S.) are most likely to experience disenfranchisement due to the criminalization of drug use, creating unique and heightened social and structural stressors affecting their mental health [18].

The impacts of healthcare and harm reduction service closures during COVID on health outcomes for WWUD have been well-documented during the pandemic, but literature about the impact of mental healthcare or harm reduction service closures in this population is scarce [19]. Mental healthcare utilization is an example of external resilience, or factors external to the individual that can support the ability to “bounce back” from hardship [20]. Qualitative research has found that high levels of poverty and other SDoH challenges can prevent WWUD harnessing external resilience by creating logistical or financial challenges to seeking mental healthcare (e.g., transportation, out-of-pocket costs) [20]. Material scarcity also creates competition for resources, undermining social support between WWUD that could form the backbone of informal mental health support [20]. Prior to the pandemic, WWUD faced substantial barriers to accessing mental healthcare and reported high levels of unmet mental health need; this is likely also true during the pandemic given wide-spread healthcare access challenges [21]. Understanding the role of unmet mental healthcare needs in the context of significant, multiple material need insecurities can provide insight into how improving access to mental healthcare may or may not mitigate mental distress for WWUD.

Both material scarcity and mental distress place WWUD at greater risk of a number of critical health-related consequences, including elevating the risk of experiencing an overdose [22, 23]. In a sample of people who use drugs in Baltimore, Maryland, unmet mental health need doubled the odds of experiencing a non-fatal overdose [15]. Further, women in the sample were at even greater risk of experiencing psychological distress than men, highlighting the importance of understanding these dynamics among WWUD in particular. While this study was conducted before the pandemic, there is also evidence that women were at greater risk of experiencing a non-fatal overdose during COVID than men [13]. Data from eight cohorts of PWUD throughout the U.S. signaled that day-to-day disruptions due to COVID were associated with 2.4 times greater odds of experiencing a recent non-fatal overdose, but specific disruptions related to material needs or other SDoH were not queried. Results from this study found that women were 2.2 times more likely than men to have experienced a non-fatal overdose.

Few empirical studies have explicitly looked at the relationship between mental health and stress-related impacts of COVID-19, such as experiencing material need challenges during the pandemic among this community in particular. We aim to characterize material need insecurities among a sample of WWUD in a large U.S. city during the COVID pandemic and to determine the unique associations between multiple material needs, unmet mental health need, and level of mental distress. Understanding changes to material need insecurities during the pandemic can have important implications for future public health preparedness and safety nets for the most marginalized populations. Studying social and economic vulnerability experienced during the pandemic offers insight into the need to attend to the social and political factors that worsen health among marginalized communities. A broader understanding of the mechanisms contributing to elevated mental distress in this population can also contribute to our understanding of the more distal sequelae of multiple material need insecurities.

## Methods

Data were collected between August 2021-December 2022 as part of the COVID Action Research Engagement (CARE) study of WWUD in Baltimore, Maryland and their experiences with COVID testing and vaccination, and the pandemic’s impact on different aspects of their lives. We used several methods to determine recruitment areas: we conducted secondary geospatial analysis of 911 calls mentioning drugs or drug use to identify areas and times of high drug activity; engaging service providers and other research teams working with this population in Baltimore to understand the changing geography of drug use in the city; and windshield tours, where staff discreetly visited potential recruitment areas to assess street activity and presence of women over 18 years old. After these areas were identified, study staff drove a mobile van to recruitment areas to consent and enroll participants and to complete a 45–60-minute interviewer-administered survey.

To recruit participants, staff parked the study van at each area, discreetly approached anyone who appeared to be a woman at least 18 years old. and asked if they would be interested in being screened for a “women’s health study.” Those interested were then screened for eligibility inside the van. Eligibility criteria included: self-identify as a woman; ≥18 years; non-medical use of opioids, crack cocaine, and/or powdered cocaine more than three times in the past three months. Participants were given a $50 Visa gift card for remuneration and were provided a bag of harm reduction supplies and references to local resources, if desired. Further recruitment and sampling methods can be found in more detail in Tomko et al. [24]. These methods yielded a final sample size of *N* = 227. The Johns Hopkins University Bloomberg School of Public Health Institutional Review Board approved all study activities, including COVID safety protocols for data collection.

### Outcome

We measured the outcome, psychological distress, using the Kessler-6, a 6-item measure of non-specific symptoms of distress and has been widely used in research and clinical settings. Symptoms assessed included: “nervous,” “hopeless,” “restless or fidgety,” “so depressed that nothing could cheer you up,” “that everything was an effort,” and “worthless.” Responses were measured on a five-point Likert scale ranging from none of the time (scored as 0) to all of the time (scored as 4) (possible range 0–24). Items were summed and scores were categorized based on validated cut-points for no or mild, moderate (*≥* 5), or severe (*≥* 13) symptoms [25].

### Exposures of interest

Material needs challenges during COVID were measured using an adapted version of the Detroit Metro Area Communities COVID-19 Survey items of pandemic-related challenges [26]. The present analysis focuses on the following five material needs: (1) housing; (2) food security; (3) access to clean, potable water; (4) transportation access; and (5) bathroom facility access. Participants indicated whether each was not a challenge, a minor challenge, or a major challenge. Each item was dichotomized into no/minor challenge versus major challenge to understand the impact of the most extreme difficulties. We determined a material need insecurities score by summing the number of major challenges reported. We dichotomized the variable as minimal (0–1) and multiple insecurities [2–5] for two reasons: to empirically explore multiple material need insecurities per the Precarity in Health framework; and there was no significant unadjusted difference between the outcome and zero or one challenge, supporting their combination (data not shown).

Mental health need was assessed by the following question: “Was there a time in the past 3 months when you needed mental health treatment but could not get it?” Responses included “no, never needed it,” “yes, but I received it,” and “yes, but did not receive it.” We dichotomized responses into “don’t need or met need” and “unmet need.”

### Covariates

Socio-demographics assessed included age (dichotomized as < 40 and *≥* 40 years), race (dichotomized as non-Hispanic white and non-Hispanic Black or person of color), being in a relationship or married, education, experiencing homelessness before the pandemic, and recent (past 3 month) history of being arrested, selling or exchanging sex, and perception of safety at home. Financial characteristics included self-reported income decrease during the pandemic and use of expanded safety net programs including Supplemental Nutrition Assistance Program (SNAP), receipt of a stimulus payment from the federal government, and eligibility for and receipt of unemployment assistance under expanded eligibility criteria during COVID. We assessed self-reported history of mental illness, including specific diagnosis. From the K-6, we asked frequency of physical health problems contributing to psychological distress; responses ranged from “none of the time” to “all the time” and we dichotomized responses as no (i.e., none of the time) versus yes (i.e., any other frequency).

Recent (past 3 month) substance use variables included any drug injection; non-medical use of: opioids (including fentanyl specifically), stimulants, or prescription pain relievers; at least daily use of any of these drugs; alcohol use at least monthly; and experience of non-fatal overdose.

### Statistical analysis

Pearson’s χ^2^ tests were used to examine bivariate differences between level of psychological distress and potential covariates. Unadjusted multinomial regressions with robust variances were used to model associations with exposures of interest and level of psychological distress, producing risk ratios (RR) and robust confidence intervals (CI). To determine covariates included in the adjusted model, we included material need insecurities and unmet mental health need in the model and retained any covariate with *p* < 0.20 in Pearson’s χ^2^ tests, then used a manual backward stepwise deletion process to remove variables. Covariates significant at *p* < 0.15 in at least one comparison in the adjusted multinomial model were retained for the final model. Significance was set at alpha < 0.05. All analyses were conducted in Stata/SE 15.1 (College Station, TX). Data is available upon reasonable request from the corresponding author.

## Results

In this sample of WWUD, 36% (*n* = 81) reported symptoms of moderate psychological distress while 39% (*n* = 89) reported symptoms of severe distress.

Socio-demographic and financial characteristics that differed significantly by level of psychological distress included race (*p* = 0.03), experiencing homelessness pre-pandemic (*p* = 0.04), recent history of selling or exchanging sex (*p* = 0.03), perception of safety in home (*p* = 0.01), and decreased income during the pandemic (*p* = 0.02) (Table 1). Over three-quarters (67%) of the sample had ever been diagnosed with a mental illness, which differed significantly by level of psychological distress (*p* < 0.001). The most frequently reported diagnosed mental illnesses included major depressive disorder (72%), bipolar depression (54%), and anxiety-related disorder (46%). Level of psychological distress also significantly differed by recent drug injection (*p* = 0.004), stimulant (*p* = 0.01) or fentanyl (*p* = 0.05) use, and non-fatal overdose (*p* = 0.03).

**Table 1:** Baseline characteristics stratified by level of psychological distress among the CARE cohort of women who use drugs (*N* = 227) in Baltimore, MD

			Psychological distress (K6)			
		Total *N* = 227	No or mild distress *N* = 57	Moderate distress *N* = 81	Severe distress *N* = 89	*p*
		N (%)	N (col %)	N (col %)	N (col %)	
Age						
	*< 40 years*	117 (51.5)	27 (47.4)	37 (45.7)	53 (59.6)	0.15
	*≥ 40 years*	110 (48.5)	30 (52.6)	44 (54.3)	36 (40.4)	
Race						
	*Black or Person of Color*	84 (40.6)	17 (32.7)	25 (33.8)	42 (51.9)	0.03
	*White*	123 (59.4)	35 (67.3)	49 (66.2)	39 (48.1)	
In a relationship or married		59 (26.8)	16 (28.6)	18 (22.8)	25 (29.4)	0.60
Education						
	*Less than high school graduate*	93 (41.3)	28 (49.1)	30 (37)	35 (40.2)	0.15
	*High school graduate or GED*	77 (34.2)	22 (38.6)	29 (35.8)	26 (29.9)	
	*At least some college*	55 (24.4)	7 (12.3)	22 (27.2)	26 (29.9)	
Experienced homelessness pre-pandemic		53 (24.1)	8 (14.3)	17 (21.8)	28 (32.6)	0.04
Arrested*		40 (17.6)	7 (12.3)	14 (17.3)	19 (21.3)	0.37
Sold or exchanged sex*		120 (55.0)	23 (42.6)	42 (52.5)	55 (65.5)	0.03
Feel safe in home*		186 (83.8)	53 (93)	70 (87.5)	63 (74.1)	0.01
Income decreased during pandemic		99 (44.2)	17 (29.8)	35 (43.8)	47 (54.0)	0.02
Expanded safety net						
	SNAP enrollment	156 (70.0)	40 (70.2)	58 (71.6)	58 (68.2)	0.89
	Received stimulus payment	113 (50.7)	32 (56.1)	42 (51.9)	39 (45.9)	0.47
	Eligible for and received unemployment	31 (13.9)	6 (10.5)	9 (11.1)	16 (18.8)	0.25
Diagnosed with mental illness		149 (67.4)	24 (42.1)	51 (63.0)	74 (89.2)	< 0.001
	PTSD^†^	39 (26.7)	3 (13)	6 (12.2)	30 (40.5)	< 0.001
	Major depressive disorder^†^	105 (71.9)	12 (52.2)	34 (69.4)	59 (79.7)	0.03
	Bipolar depression^†^	79 (54.1)	12 (52.2)	23 (46.9)	44 (59.5)	0.39
	Schizophrenia^†^	19 (13.0)	2 (8.7)	7 (14.3)	10 (13.5)	0.79
	Anxiety-related disorder^†^	67 (45.9)	7 (30.4)	21 (42.9)	39 (52.7)	0.15
Physical health affecting mental health		105 (46.3)	26 (45.6)	35 (43.1)	44 (49.4)	0.71
Substance use*						
	Injected any drug	79 (34.8)	18 (31.6)	19 (23.5)	42 (47.2)	0.004
	Daily use of any drug	194 (85.5)	48 (84.2)	70 (86.4)	76 (85.4)	0.94
	Opioid use	191 (90.1)	47 (85.5)	67 (90.5)	77 (92.8)	0.37
	Simulant use	199 (92.6)	44 (83)	76 (95)	79 (96.3)	0.01
	Fentanyl use	130 (67.0)	32 (61.5)	40 (59.7)	58 (77.3)	0.05
	Non-medical prescription pain reliever use	19 (10.8)	1 (2.1)	9 (13.9)	9 (14.3)	0.07
	Any alcohol use at least monthly	53 (23.7)	13 (22.8)	19 (23.5)	21 (24.4)	0.97
Non-fatal overdose*		52 (23.3)	11 (19.3)	13 (16.1)	28 (32.9)	0.03
Health insurance		205 (92.3)	50 (87.7)	76 (95.0)	79 (92.9)	0.28
Has regular physician		142 (63.4)	39 (68.4)	57 (70.4)	46 (53.5)	0.05
Has regular clinic they visit		147 (65.6)	40 (70.2)	57 (70.4)	50 (58.1)	0.18

* Past 3 months

^†^ Of *N* = 146 diagnosed with mental illness

Prevalence of each of the individual major material need insecurities ranged from 23 to 38% and 43% of WWUD reported multiple material need insecurities during COVID (Table 2).

**Table 2:** Unadjusted associations between material need insecurities during COVID, unmet mental health need, and level of mental distress among women who use drugs (*N* = 227) in Baltimore, Maryland

		No or mild vs. severe		No or mild vs. moderate		Moderate vs. severe	
	N (%)	Risk Ratio (95% CI)	*p*	Risk Ratio (95% CI)	*p*	Risk Ratio (95% CI)	*p*
Housing							
*Not/minor challenge*	139 (62.1)	*Reference*		*Reference*		*Reference*	
*Major challenge*	85 (37.9)	**7.04 (2.98**,** 16.66)**	**< 0.001**	**3.80 (1.59**,** 9.10)**	**0.003**	1.86 (1.00, 3.44)	0.05
Food security							
*Not/minor challenge*	146 (65.2)	*Reference*		*Reference*		*Reference*	
*Major challenge*	78 (34.8)	**2.68 (1.26**,** 5.69)**	**0.01**	1.69 (0.78, 3.67)	0.18	1.58 (0.84, 2.97)	0.15
Access to clean, potable water							
*Not/minor challenge*	172 (76.8)	*Reference*		*Reference*		*Reference*	
*Major challenge*	52 (23.2)	2.31 (0.99, 5.41)	0.05	1.42 (0.58, 3.46)	0.44	1.63 (0.80, 3.31)	0.18
Transportation access							
*Not/minor challenge*	139 (62.1)	*Reference*		*Reference*		*Reference*	
*Major challenge*	85 (37.9)	**2.93 (1.40**,** 6.13)**	**0.004**	1.71 (0.80, 3.65)	0.16	1.71 (0.92, 3.19)	0.09
Bathroom facility access							
*Not/minor challenge*	159 (71.0)	*Reference*		*Reference*		*Reference*	
*Major challenge*	65 (29.0)	**6.42 (2.48**,** 16.59)**	**< 0.001**	**3.17 (1.19**,** 8.44)**	**0.02**	**2.03 (1.06**,** 3.88)**	**0.03**
Cumulative material need insecurities							
*Minimal (0–1)*	130 (57.3)	*Reference*		*Reference*		*Reference*	
*Multiple (2–5)*	97 (42.7)	**3.94 (1.90**,** 8.14)**	**< 0.001**	1.65 (0.78, 3.47)	0.19	**2.39 (1.29**,** 4.44)**	**0.01**
Mental health need							
*Don’t need or met need*	142 (62.6)	*Reference*		*Reference*		*Reference*	
*Unmet need*	85 (37.4)	**4.54 (2.15**,** 9.61)**	**< 0.001**	1.19 (0.54, 2.62)	0.68	**3.84 (2.00**,** 7.36)**	**< 0.001**

Several individual insecurities and cumulative material need insecurities were significantly associated with elevated psychological distress in unadjusted models. Compared to no or mild distress, participants were more likely to have severe distress if they experienced major insecurities in housing (RR = 7.04, 95% CI = 2.98–16.66), food security (RR = 2.68, 95% CI = 1.26–5.69), transportation (RR = 2.93, 95% CI = 1.40–6.13), bathroom access (RR = 6.42, 95% CI = 2.48–16.59), and multiple material needs challenges (RR = 3.94, 95% CI = 1.90–8.14). Compared to no or mild distress, participants were more likely to have moderate distress if they experienced major insecurities in housing (RR = 3.80, 95% CI = 1.59–9.10) and bathroom access (RR = 3.17, 95% CI = 1.19–8.44). Finally, compared to moderate distress, participants were more likely to have severe distress if they experienced a major challenge in bathroom access (RR = 2.03, 95% CI = 1.06–3.88) and had multiple material need insecurities (RR = 2.39, 95% CI = 1.29–4.44).

Compared to those that do not need or met their mental health need, participants with unmet mental health need were significantly more likely to have severe psychological distress compared to those with no/mild distress (RR = 4.54, 95% CI = 2.15–9.61) or moderate distress (RR = 3.84, 95% CI = 2.00-7.36).

An adjusted multinomial model showed significant relationships between key exposures and level of distress (Table 3).

**Table 3:** Adjusted multinomial regression comparing levels of mental distress in a sample of women who use drugs in Baltimore, Maryland (*n* = 227)

	No or mild vs. severe		No or mild vs. moderate		Moderate vs. severe	
	Adjusted* Risk Ratio (95% CI)	*p*	Adjusted* Risk Ratio (95% CI)	*p*	Adjusted* Risk Ratio (95% CI)	*p*
Cumulative material need insecurities						
*Minimal (0–1)*	*Reference*		*Reference*		*Reference*	
*Multiple (2–5)*	**3.25 (1.42**,** 7.45)**	**0.01**	1.66 (0.77, 3.56)	0.19	1.96 (0.97, 3.95)	0.06
Mental health need						
*Don’t need or met need*	*Reference*		*Reference*		*Reference*	
*Unmet need*	**3.44 (1.48**,** 7.97)**	**0.004**	0.95 (0.41, 2.18)	0.90	**3.62 (1.75**,** 7.49)**	**0.001**
Injected drugs						
*No*	*Reference*		*Reference*		*Reference*	
*Yes*	1.95 (0.85, 4.47)	0.12	0.65 (0.30, 1.39)	0.27	**3.01 (1.41**,** 6.45)**	**0.004**
Diagnosed with mental illness						
*No*	*Reference*		*Reference*		*Reference*	
*Yes*	**9.14 (3.61**,** 23.14)**	**< 0.001**	**2.30 (1.14**,** 4.67)**	**0.02**	**3.97 (1.63**,** 9.66)**	**0.002**

Compared to those who reported minimal material need insecurities, participants with multiple insecurities were 3.25 (95% CI = 1.42–7.45) and 1.96 (95% CI = 0.97–3.95) times more likely to report severe psychological distress compared to no/mild distress or moderate distress, respectively. Similarly, compared to those with their mental health needs met, those with unmet need were 3.44 (95% CI = 1.48–7.97) and 3.62 (95% CI = 1.75–7.49) times more likely to report severe psychological distress compared to no/mild distress or moderate distress, respectively. Recent drug injection was associated with a three-fold increase (aRR = 3.01, 95% CI = 1.41–6.45) in risk of severe mental distress compared to moderate distress. History of mental illness diagnosis was significantly associated with greater likelihood of severe distress compared to no/mild distress (aRR = 9.14, 95% CI = 3.61–23.14) or moderate distress (aRR = 3.97, 95% CI = 1.63–9.66). Participants with history of mental illness history were more than twice as likely to have moderate distress compared to no/mild distress (aRR = 2.30, 95% CI = 1.14–4.67).

## Discussion

This study of COVID-era material need insecurities among WWUD in Baltimore, Maryland revealed a substantial burden of challenges to meeting basic needs of housing, food, or water access during the pandemic. These insecurities were associated with elevated risk of experiencing severe mental distress compared to mild or moderate distress even when controlling for other threats to psychological well-being, finding support for the Precarity in Health framework and underscoring the upstream role that SDoH play in mental health. Results speak to the need to better address access to the SDoH and mental healthcare for marginalized populations, particularly during health emergencies that threaten already-precarious social safety nets and healthcare infrastructure. Results further speak to the need to broadly reframe mental health from an individual concern to public mental health, recognizing social and economic policies that can impact population-wide mental health outcomes.

It is striking that, for every material need challenge assessed, at least one-quarter of the women in the study reported it to be a major challenge during COVID. Perhaps even more notable is that, cumulatively, nearly half the sample reported experiencing multiple material needs during COVID. Multinomial models showed the role that experiencing multiple material need insecurities plays in distinguishing those with severe mental distress from lesser levels of distress. In unadjusted models, nearly all individual material needs were associated with greater odds of severe mental distress compared to no mental distress. However, there were few individual material needs significantly associated with severe mental distress compared to moderate distress; rather, only experiencing cumulative multiple material need insecurities was associated with greatest levels of distress. Taken together, these results suggest ways that addressing material needs may improve mental distress levels among WWUD. For individuals not experiencing distress, improving access to any material need may have a protective effect on mental health and prevent future severe mental distress. Yet for those already experiencing moderate distress, it may be more important to understand and address cumulative, multiple material needs than any one need in particular.

Our research assumes material needs exist prior to and have an effect on mental distress [5]. It is important to acknowledge that the relationship between material needs and mental distress may be bidirectional and pre-existing mental distress prior to the pandemic may have contributed to more serious material needs when the pandemic hit. Regardless of the directionality of the relationship, both scenarios suggest a problematic social safety net in the US that fails to adequately bolster SDoH and mental healthcare in times of emergency or crisis [5]. Just about half of the sample reported receiving a stimulus payment during COVID and only 14% were eligible for and received unemployment assistance, despite the sample experiencing demonstrable financial hardships. Further, measures of an expanded safety net during COVID were not significantly different between levels of mental distress in bivariate or multivariable models. Existing social programs administered by the US federal government have been critiqued for their burdensome enrollment process and inadequate benefits. For example, stimulus payment and expanded eligibility requirements for unemployment during COVID provided some relief for low-income individuals but were critiqued for being temporary, an insufficient amount to meet the magnitude of needs during the pandemic, and inequitably distributed [27]. Strengthening the social safety net for WWUD may include increasing support for WWUD navigating applications for future temporary cash relief such as stimulus payments or SNAP, for example, or permanently broadening eligibility requirements for unemployment benefits [28]. Universal basic income and single-payer healthcare coverage are two policy examples cited as potential national-level interventions to improve poor mental health via addressing the SDoH [29, 30]. Federal and state laws in the U.S. should also consider less punitive drug policies that restrict formal employment opportunities and enrollment in safety net benefits, leading to systemic disenfranchisement of WWUD.

This study was conducted in Baltimore, Maryland, a state in the US with a relatively well-performing economy, in one of the highest-income countries in the world. To our knowledge, there are very few studies of the effects of changes in material needs during COVID and mental health among marginalized populations in high income countries [31, 32]. There is a growing recognition of the affordable housing and food insecurity crises in the US and other high-income countries; previously, the impact of these crises had largely been established in middle- or lower-income countries. There is even less recognition of difficulties of residents of a high-income country accessing clean water or a bathroom to wash, especially as it pertains to already-marginalized populations such as WWUD. The dual stigmas of substance use and poverty often render the hardships experienced by these populations forgotten or unimportant, a result of their own personal failings, and experiences of stigma are more pronounced among people enrolled in SNAP or other social programs [33]. Prior research has found heightened feelings of isolation, shame, guilt, and helplessness among people living in poverty in higher-income countries given the relative economic stability of the population at large, contributing to unique impacts on mental distress in this context [34, 35]. Structural stigmas and the institutions that perpetuate them against WWUD require policy-driven solutions to make broader systemic changes that address economic precarity in such populations, though the retraction of the welfare state in Eastern Europe and North America means that changing macro-economic trends and political norms are prerequisites for the necessary structural solutions.

Unmet mental health need also distinguishes participants with severe distress from lesser levels of distress. Results showed a three-fold increase in odds of experiencing elevated mental distress for participants experiencing unmet mental health needs. Relatedly, participants with a diagnosed mental illness were also more likely to experience severe distress compared to no/mild or moderate distress. History of mental illness does not dictate that individuals must live with mental distress; symptoms of distress can be well-managed provided that consistent access to care can be maintained. Yet results also show that mental healthcare alone is insufficient to address mental health in this population without addressing material need insecurities. WWUD have described many overlapping competing priorities creating barriers to accessing mental healthcare including the demands of substance use and drug withdrawal [36]. Earning money through whatever means to meet basic needs such as housing, food, and water are also oft-cited competing priorities that take a backseat to accessing mental healthcare, even in times of significant distress, and can also undermine social support among WWUD that could be a source of informal support.

These results are subject to several important limitations. First, the sample size of this study is modest and, as such, results that do not meet the threshold for statistical significance here may in fact be significant correlates with more statistical power. Second, we collapsed our primary exposure variable, material need challenges during COVID, into a binary variable representing the most severe material deprivation. Therefore, results cannot speak to the mental health impacts of lesser levels of material deprivation should they exist. Third, we did not have a measure of material needs or mental health pre-pandemic and therefore cannot say that the significant relationships described are caused by conditions solely during the pandemic. Fourth, these findings may be subject to bias in who self-selected into study participation and in the self-reported nature of survey data. Finally, we are limited by the specific measures of mental distress and material need challenges. For example, the Kessler-6 is a non-specific measure of mental distress so it is not possible to draw conclusions about particular relationships between material needs and specific mental health symptoms. Further, the Whittle framework provides a useful basis upon which to re-conceptualize the dimensions of material needs beyond extreme deprivation, but our data are not able to assess quality, uncertainty, or social acceptability of needs. Future work can use more specific measures of mental health symptoms and assess participants’ perceptions of the multidimensionality of material needs.

## Conclusion

In a study of women who use drugs in Baltimore, Maryland, results showed substantial challenges to meeting basic needs like housing, food, clean water, and bathroom access during the COVID-19 pandemic, with nearly half the sample reported more than one material need insecurity during this time. Over one-third of the sample reported severe mental distress, greater risk for which was significantly associated with multiple material need insecurities compared to mild or moderate distress, and which itself confers additional risk for overdose and other drug-related harms. Results support the idea that material need insecurities—part of the social determinants of health—play a critical role in mental health outcomes in economically and socially marginalized populations. Results also emphasize the need to address access to the social determinants of health and mental healthcare for marginalized populations, especially during health emergencies that strain social safety nets and healthcare infrastructure.

## Data Availability

Data is available upon reasonable request from the corresponding author.
